# Aberrant accumulation of age- and disease-associated factors following neural probe implantation in a mouse model of Alzheimer’s disease

**DOI:** 10.1088/1741-2552/aceca5

**Published:** 2023-09-01

**Authors:** Steven M Wellman, Olivia A Coyne, Madeline M Douglas, Takashi D Y Kozai

**Affiliations:** 1 Department of Bioengineering, University of Pittsburgh, Pittsburgh, PA, United States of America; 2 Center for Neural Basis of Cognition, Pittsburgh, PA, United States of America; 3 Center for Neuroscience, University of Pittsburgh, Pittsburgh, PA, United States of America; 4 McGowan Institute of Regenerative Medicine, University of Pittsburgh, Pittsburgh, PA, United States of America; 5 NeuroTech Center, University of Pittsburgh Brain Institute, Pittsburgh, PA, United States of America

**Keywords:** lysosome, metabolism, foreign body response, neural interfaces, amyloid beta, tauopathy

## Abstract

*Objective*. Electrical stimulation has had a profound impact on our current understanding of nervous system physiology and provided viable clinical options for addressing neurological dysfunction within the brain. Unfortunately, the brain’s immune suppression of indwelling microelectrodes currently presents a major roadblock in the long-term application of neural recording and stimulating devices. In some ways, brain trauma induced by penetrating microelectrodes produces similar neuropathology as debilitating brain diseases, such as Alzheimer’s disease (AD), while also suffering from end-stage neuron loss and tissue degeneration. The goal of the present study was to understand whether there may be any parallel mechanisms at play between brain injury from chronic microelectrode implantation and those of neurodegenerative disorder. *Approach*. We used two-photon microscopy to visualize the accumulation, if any, of age- and disease-associated factors around chronically implanted electrodes in both young and aged mouse models of AD. *Main results*. We determined that electrode injury leads to aberrant accumulation of lipofuscin, an age-related pigment, in wild-type and AD mice alike. Furthermore, we reveal that chronic microelectrode implantation reduces the growth of pre-existing Alzheimer’s plaques while simultaneously elevating amyloid burden at the electrode-tissue interface. Lastly, we uncover novel spatial and temporal patterns of glial reactivity, axonal and myelin pathology, and neurodegeneration related to neurodegenerative disease around chronically implanted microelectrodes. *Significance*. This study offers multiple novel perspectives on the possible neurodegenerative mechanisms afflicting chronic brain implants, spurring new potential avenues of neuroscience investigation and design of more targeted therapies for improving neural device biocompatibility and treatment of degenerative brain disease.

## 1. Introduction

Penetrating microelectrodes offer investigators and clinicians a multi-modal approach for recording discrete signals from the brain and exogenously manipulating neuronal activity with high spatial and temporal resolution through stimulation[1]. Intracortical recording and manipulation of neural signals is an emerging clinical technique for diagnosing and addressing impaired brain function and treating neurological disorders [2, 3]. Yet, significant unknowns in how the presence of an indwelling foreign object alters the structure and physiology of various brain cells, and by extension the robust and reliable recording of extracellular potentials, limit the long-term clinical applications of the technology [4–7]. Furthermore, current standard operating procedures do not presently screen clinical users of implantable neural interfaces for potential history of genetic predisposition to neurodegenerative or neurological disease, presenting a significant confound toward current efforts in understanding the brain’s immune response to neural electrode implants.

Implantation of intracortical microelectrodes can have long-lasting negative effects on neuronal health and brain function. Insertion of a stiff, sharp microelectrode can immediately sever neurons, axons, and blood vessels within the parenchyma, generating mechanical strain, neuroinflammation, and cerebral bleeding [5, 8, 9]. This acute insult leads to the activation of local glial and immune cells and release of factors which promotes inflammation, excitotoxicity, and oxidative stress, compromising local metabolic support [4]. The long-term presence of an indwelling electrode inevitably results in the development of an encapsulating glial scar impermeable to the diffusion of ions and metabolites required to detect neuronal activity, which can physically displace neurons farther from the device surface and produce a neurotoxic microenvironment [5, 10]. The gradual decline in performance and fidelity of recording and stimulating electrodes is ultimately attributed to this seemingly unavoidable progression in gliosis and neurodegeneration [11]. However, attempts at identifying biological correlates of signal degradation, such as glial activation and neuronal viability, have proven unsuccessful, suggesting other biological mechanisms may be responsible for brain tissue response outcomes and long-term device performance [5].

Age-related degenerative brain diseases, such as Alzheimer’s disease (AD), culminate in late-stage neuron loss and chronic tissue inflammation as a result of presently unknown neurodegenerative mechanisms which gradually worsen over time [12, 13]. AD is characterized by the appearance of extra-synaptic amyloid beta (A*β*) plaques and intra-neuronal neurofibrillary tau tangles and is clinically diagnosed by functional impairments in cognition and behavior [14–16]. Despite several clinical trials demonstrating promising therapeutic potential at reducing A*β* and tau pathology or ameliorating clinical symptoms [13, 17], there is currently no known cure or effective treatment for AD. The inherent biological cause(s) of AD remain elusive but several genetic (e.g. *APOE, APP, TREM2*) [18–20] and non-genetic [21] (e.g. diet, exercise, cardiovascular health, and brain injury) risk factors for developing AD have been proposed. Particularly, trauma suffered to the brain has been linked to an increased risk of AD years after the initial insult and is most likely due to the nature in which brain injury and dementia share similar patterns of neurodegeneration [22–24]. The progression of AD as it is currently known shares many similarities with that of focal injury from a penetrating microelectrode array such as neuroinflammation, glial activation, and vascular injury. Hyperphosphorylated tau, a precursor for development of pathological tau tangles observed in AD, is reportedly elevated near chronically implanted microelectrodes within both wild-type rats and human Parkinsonian patients [25]. It is currently unknown, however, whether the neurotrauma suffered by intracortical microelectrodes in healthy control subjects is in any way similar to that observed in AD progression.

Characterizing A*β* deposition and tau accumulation in Alzheimer’s and other neurodegenerative diseases often require the use of genetically specialized and aged mouse models, a cost- and time-expensive process. Furthermore, under normal conditions it is often difficult to pinpoint the time and location of the biological processes responsible for the onset and progression of neurodegeneration. In contrast, focal tissue injury due to insertion of a penetrating microelectrode occurs at a known time and location within the brain, which allows for neurodegenerative processes to be spatiotemporally characterized from the onset. In this study, we investigated the appearance, if any, of age- and disease-associated factors following chronic implantation (12–16 weeks) in healthy, wild-type mice as well as a genetically susceptible mouse model of AD (APP/PS1). We hypothesized that the foreign body response to intracortical electrodes promotes the onset and progression of brain pathology typically observed within aging and degenerative brain disease, resulting in long-term neuron loss. Using *in vivo* imaging and post-mortem immunohistology, we determined that microelectrode implants exacerbate the accumulation of lipids and proteins such as lipofuscin and amyloid and promote the expression of other pathological factors associated with degenerative brain disease. In summary, our work improves our understanding of neurodegenerative processes at the intersection of brain injury and disease, which will have profound impacts on development of future intervention strategies for both implantable neural interfaces and neurological disorders within the brain.

## 2. Methods

### 2.1. Experimental animal models

C57BL/6J (2 months old and 18 months old, male, 22–30 g, strain# 664, Jackson Laboratories; Bar Harbor, ME), B6.Cg-Apoe^tm1.1^(^APOE*4^)^Adiuj^App^em1Adiuj^Trem2^em1Adiuj^/J (18 months old, male, 22–30 g, strain# 30670, Jackson Laboratories; Bar Harbor, ME), and APP/PS1 (2 months old and 6 months old, male, 22–30 g, strain# 34829, Jackson Laboratories; Bar Harbor, ME) were used in this study. All animal care and procedures were performed under approval of the University of Pittsburgh Institutional Animal Care and Use Committee and in accordance with regulations specified by the Division of Laboratory Animal Resources.

### 2.2. Probe implantation surgery

APP/PS1 mice at 2 months old (*n* = 4) or 6 months old (*n* = 3), WT mice at 18 months old (*n* = 3), and triple-mutant hAbeta/APOE4/Trem2*R47H mice at 18 months old (*n* = 3) were implanted with a four-shank Michigan style microelectrode array for awake, head-fixed 2P imaging, as described previously [5, 8, 10, 26–28]. One 18 m.o. WT mouse and one 18 m.o. hAbeta/APOE4/Trem2*R47H mouse passed within the first week following implantation surgery, mostly likely due to difficulty in performing invasive surgical procedures on aged mice, and therefore their data could not be included in the study. Briefly, mice were sedated with an anesthetic cocktail (7 mg kg^−1^ xylazine and 75 mg kg^−1^ ketamine). The surgical site was shaved and sterilized 2–3 times with alternate scrubs of an aseptic wash and 70% ethanol. Mice were then fixed to a stereotaxic frame and a small incision was made over the skull. Care was given to remove all skin, hair, and connective tissue from the surface of the skull before a thin layer of Vetbond (3 M) was applied to the surface. A rectangular head bar was fixed to the skull for awake, head-fixed imaging. Four bone screw holes were drilled and bone screws inserted over both ipsilateral and contralateral motor cortices and lateral visual cortices to secure the head bar. A 4 mm by 4 mm bilateral craniotomy was performed prior to probe insertion. The skull was periodically bathed in sterile saline to prevent the skull from overheating during drilling. Probes were inserted through an intact dura into the cortex at a 30° angle at 200 *μ*m s^−1^ for a total linear distance of ∼600 *μ*m (oil hydraulic Microdrive; MO-82, Narishige, Japan) and final z-depth of ∼250–300 *μ*m beneath the pial surface. The craniotomy was filled with sealant (Kwik-Sil) before sealing with a glass coverslip and dental cement. Anesthesia was maintained throughout the surgery with ketamine (40 mg kg^−1^), as needed. Ketofen (5 mg kg^−1^) was provided post-operatively up to two days post-surgery or as needed.

For immunohistochemistry, a separate cohort of 2 months old C57BL/6J (*n* = 6) and 2 months old APP/PS1 mice (*n* = 6) were implanted as described above with the exception of a single-shank probe implanted at a 90° angle, as described previously [7]. Briefly, a drill-bit sized craniotomy was formed over the left primary visual (V1) cortex (1.5 mm anterior and 1 mm lateral from lambda) and a Michigan-style microelectrode was inserted at a rate of 200 *μ*m s^−1^ to a final resting depth of 1600 *μ*m below the pial surface. The probe and craniotomy were filled with Kwik-sil and a headcap was secured with dental cement.

### 2.3. Two-photon imaging and plaque labeling

Two-photon microscopy was used to track the rate of amyloid deposition or clearance around implanted microelectrodes in 6 m.o. WT and APP mice or 18 m.o. WT and hAbeta/APOE4/Trem2*R47H over a 12–16 week implantation period (0, 2, 4, 7, 14, 21, 28, 56, 84, and 112 d post-implantation), as described previously [8, 10, 26, 27]. The microscope was equipped with a scan head (Bruker, Madison, WI), a OPO laser (Insight DS+; Spectra-Physics, Menlo Park, CA), non-descanned photomultiplier tubes (Hamamatsu Photonics KK, Hamamatsy, Shizuoka, Japan), and a 16X, 0.8 numerical aperture water-immersive objective lens (Nikon Instruments, Melville, NY). To visualize plaques, mice were injected intraperitoneally with methoxy-X04 (2 mg kg^−1^, Abcam, #ab142818) 24 h prior to each imaging session[20]. Mice were retro-orbitally injected with FITC-dextran (2 MDa, 0.03 ml at 10 mg ml^−1^) immediately prior to imaging to visualize surrounding blood vessels. The vasculature was used as a landmark to ensure similar ROI fields were captured between subsequent imaging sessions. Methoxy-X04 was excited at a 740 nm laser excitation wavelength and care was given not to exceed >20–30 mW of power during chronic imaging. Z-stacks were collected along the full depth of the implant at a step size of 2–3 *µ*m, ∼5 s frame rate, and zoom factor of ∼1.5-2x.

### 2.4. Immunohistochemical staining

C57BL/6J and APP/PS1 mice that were implanted at ages of 2 months old were sacrificed and perfused according to University of Pittsburgh IACUC approved methods at 1 week (*n*= 6 per group) or 16 weeks (*n* = 7 per group) post-implantation, as described previously [7]. Briefly, mice were sedated using a cocktail mixture of xylazine (7 mg kg^−1^) and ketamine (75 mg kg^−1^). A toe-pinch test was performed to ensure proper level of anesthesia prior to beginning the procedure. For each mouse, 100 ml of warm phosphate buffered saline (PBS) was perfused transcardially (pump pressure between 80–100 mm Hg) followed by 100 ml of 4% paraformaldehyde (PFA). Mice were then decapitated with both skull and implant left intact for 24 h post-fixation at 4 °C in 4% PFA. This extended fixation period ensured the tissue surrounding the microelectrode remains minimally disturbed during implant removal. Brains were then carefully detached from the skull by removing excess bone and gently pushing down on the headcap while the tissue sample is inverted, allowing the probe to fall straight down out of the brain. Brain samples were then sequentially soaked in 15% and 30% sucrose in PBS at 4 °C for 24 h each. Once the tissue had reached sucrose equilibration, they were frozen in a 2:1 ratio of 20% sucrose:optimal cutting temperature compound (Tissue Tek, Miles Inc., Elkhart, IN, United States). Frozen brain samples were then sectioned horizontally using a cryostat (Leica Biosystems, Wetzlar, Germany) at a 25 *μ*m thickness throughout the entire depth of the implant (∼1600 *μ*m).

Brain tissue sections between 150–300 *μ*m cortical depth (layer II/III) were chosen for immunohistochemical analysis, corresponding with the depth of observation during 2P imaging. Before staining, frozen tissue sections were re-hydrated with two washes of 1x PBS for 5 min each. For antigen retrieval, slides were incubated in 0.01 M sodium citrate buffer for 30 min at 60 °C. Slides were then incubated in in a peroxidase blocking solution (10% v/v methanol and 3% v/v hydrogen peroxide in 1x PBS) for 20 min on a table shaker (60 r.p.m.) at room temperature (RT) to block for active aldehydes and reduce the chance of non-specific binding. Sections were then pre-treated with a solution of 1% triton X-100 and 10% donkey serum in 1x PBS for 1 h at RT followed by blocking endogenous mouse immunoglobulin G (IgG) with donkey anti-mouse IgG fragment (Fab) for 2 h at 1:10 dilution at RT. Then, sections were rinsed with alternating washes of 1x PBS and 1x PBS-T (1% v/v of Tween-20 in 1x PBS) for four times 4 min each. Primary antibodies were diluted in solution of 1% triton X-100 and 10% donkey serum and applied to slides for 12–18 h at 4 °C. All primary antibodies used in this study are listed in table 1. Sections were rinsed in 3 × 5 min washes of 1x PBS the following day. Secondary antibodies were diluted 1:500 in 1x PBS and applied to slides for 2 h at RT. Secondary antibodies used were: Alexa Fluor 488 donkey anti-mouse, Alexa Fluor 488 donkey anti-rabbit, Alexa Fluor 568 donkey anti-mouse, Alexa Fluor 568 donkey anti-rat, Alexa Fluor 568 donkey anti-goat, Alexa Fluor 568 donkey anti-sheep, Alexa Fluor 647 donkey anti-mouse, Alexa Fluor 647 donkey anti-chicken, and Alexa Fluor 647 streptavidin (Abcam, Cambridge, UK). Then, slides were rinsed once more with 3 × 5 min washes of 1x PBS. To stain for cell nuclei, slides were incubated in Hoechst 33 342 (Invitrogen) at 1:1000 in 1x PBS for 10 min at RT followed by another rinse 3 × 5 min with 1x PBS. Finally, slides were cover slipped using Fluoromount-G (Southern Biotech, Birmingham, AL, United States) and sealed prior to imaging.

**Table 1.** List of primary antibodies used for immunohistochemical staining.

Primary antibody	Target	Supplier	Host	Dilution
Anti-6E10	Amyloid beta	Enzo Life Sciences (ABS612)	Rb	1:200
Anti-APP	Amyloid precursor protein	Abcam (ab2084)	Gt	1:400
Anti-AT8	Phospho-tau	Thermo Fisher (MN1020B)	Biotin	1:250
Anti-GFAP	Astrocytes	Abcam (ab4674)	Ck	1:500
Anti-Iba1	Microglia	Sigma (MABN92)	Ms	1:400
Anti-Myelin Basic Protein	Myelin	Abcam (ab7349)	Rt	1:100
Anti-NeuN	Neurons	Thermo Fisher (MA5-33103)	Ms	1:500
Anti-NF200	Neurofilament	Sigma (N5389)	Ms	1:250
Anti-Trem2	Triggering receptor expressed on myeloid cells 2	R&D Systems (AF1729)	Sh	1:100

Samples were imaged using a confocal microscope (FluoView 1000, Olympus, Inc. Tokyo, Japan) using a 20x oil-immersive objective lens (Nikon Instruments, Melville, NY). For each section, an ipsilateral and contralateral image was captured using same laser intensity and image settings for data normalization. For each image, a z-stack was collected consisting of six image planes each spaced 5 *μ*m apart (635.9 × 635.9 *μ*m, 1024 × 1024 pixels on FV10-ASW Viewer V4.2). Raw images were saved as 16-bit grayscale TIFFs.

### 2.5. Data analysis

For two-photon quantification, the distance between lipofuscin granules, plaques, or clusters and the implant were determined by referencing to a 3D distance map of the electrode surface, as described previously [5]. First, a 2D mask of the electrode footprint was manually outlined within ImageJ (National Institute of Health). Considering the original electrode implantation angle of 25–30° this 2D electrode mask was digitally rotated using ImageJ’s built-in ‘Interactive Stack Rotation’ tool. A distance map was then generated by applying a distance transform to this 3D mask using the ‘bwdist’ function in Matlab (MathWorks, Boston, MA). The spatial coordinates of segmented lipofuscin, plaques, or clusters were cross-referenced with this 3D distance map to determine the nearest Euclidean distance to the electrode surface for each timepoint.

For lipofuscin quantification, lipofuscin granules were identified by their natural autofluorescence signal detected across multiple emission filters (595 nm for green and 525 nm for red, 50 nm bandpass). First, multi-channel z-stack images were spectrally unmixed using the ImageJ plugin, ‘LUMoS Spectral Unmixing’. Then, background noise was subtracted by applying a Gaussian filter (sigma = 20) to the unmixed image containing the lipofuscin signal and subtracting the filtered image from the original. Background was further reduced by applying a bandpass filter, followed by a median filter, then finally using ImageJ’s ‘Despeckle’ function. The resultant image was binarized by applying a fluorescence intensity threshold (mean + 2.5 standard deviation) before running the ‘3D Objects Counter’ plugin to determine the size and spatial coordinates of segmented lipofuscin granules. For plaque quantification, images with methoxy-X04 signal were processed similarly as described above. The spatial coordinates of both amyloid clusters and plaques were referenced to respective 3D electrode distance map for each timepoint. The rate of change in size of individual plaques were tracked longitudinally by referencing with the vasculature as a landmark. Finally, quantification of amyloid plaques stained with 6E10 were performed using a similar ImageJ plug-in described above with the exception of using 2D confocal images as the input image in ImageJ instead of 3D Z-stacks.

For fluorescence intensity analyses of stained tissue, images were processed using a semi-automated intensity-based custom Matlab script (INTENSITY Analyzer), as described previously [7, 29]. Briefly, images were binned concentrically every 10 *μ*m up to 300 *μ*m away from the probe center. The average grayscale intensity was calculated for every pixel above a threshold determined from the intensity of the background noise for each bin. Fluorescence intensities were then normalized using average intensity values collected from the contralateral hemisphere of the section (i.e. intact hemisphere). For cell counting of stained tissue, images were binned concentrically every 50 *μ*m up to 300 *μ*m away from the probe center. Cells were identified and counted based on the presence of DAPI-stained nuclei and data was similarly normalized using cell counts from the contralateral hemisphere. Normalized intensity and cell counts were average over all animals per timepoint per group and presented as mean ± standard error.

### 2.6. Statistics

A two-way repeated measures ANOVA (*p* < 0.05) was used to determine significant differences in changes in plaque volume, staining fluorescence intensities, and histological cell counts between WT and APP/PS1 mice followed by a post-hoc *t*-test with a Bonferroni correction to determine pairwise significances between groups. A Welch’s *t*-test (*p* < 0.05) was used to determine significant differences in 6E10 staining plaque area.

## 3. Results

**Figure 1.**
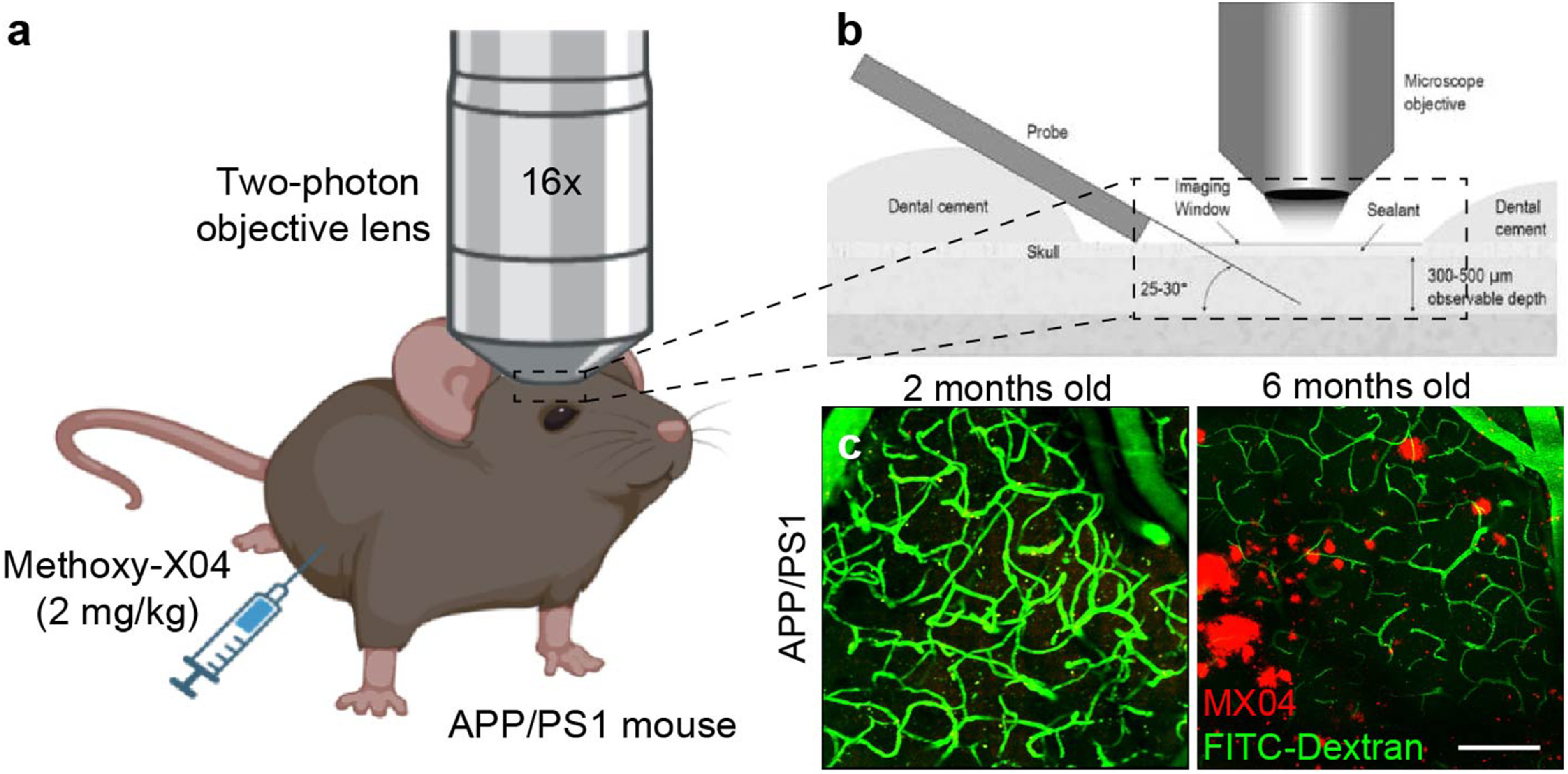
Two-photon visualization of age- and disease-related factors in a mouse model of Alzheimer’s disease. (a) Methoxy-X04 (2 mg kg^−1^) was administered to APP/PS1 mice in order to visualize Alzheimer’s plaque deposition within the mouse brain using two-photon microscopy. (b) Schematic of microelectrode implantation within the mouse cortex underneath an optical window for longitudinal imaging of chronic tissue response. (c) Representative two-photon image demonstrating age-dependency of plaque visualization (MX04, *red*) in APP/PS1 mice. Vasculature is labeled with FITC-dextran (*green*). Scale bar = 100 *μ*m.

Unlike humans, rodents do not develop Alzheimer’s pathology naturally with aging. Therefore, specially developed transgenic mouse models are required to study the progression of neuropathology within AD [30]. Here, we use the APP/PS1 mouse model expressing a humanized form of the *APP* gene, which promotes overexpression of the amyloid precursor protein (APP), as well as the *PSEN1* gene, which controls for expression of the enzyme presenilin 1 required for proteolytic cleavage of amyloid (figure 1(a)). These mice have been extensively characterized for study of AD and therefore their temporal onset and progression of amyloid pathology is well known [31]. To determine whether chronic electrode implantation leads to accumulation of age- or disease-associated factors, we used two-photon microscopy to longitudinally quantify aberrant protein aggregation around a multi-shank Michigan style microelectrode array within the WT and AD mouse cortex (figure 1(b)). Specifically, to assess age-related lipofuscin accumulation, mice were aged to 18 months prior to electrode implantation surgery. Next, to assess for plaque deposition, young (2-month-old) and adult (6-month-old) APP/PS1 mice were intraperitoneally administered methoxy-X04 (MX04), a Congo-red derivative which binds to amyloid and tau [32], 24 h prior to each imaging session (figure 1(c)).

### 3.1. Aberrant accumulation of lipofuscin around chronically implanted electrodes in aged WT and AD mice

**Figure 2.**
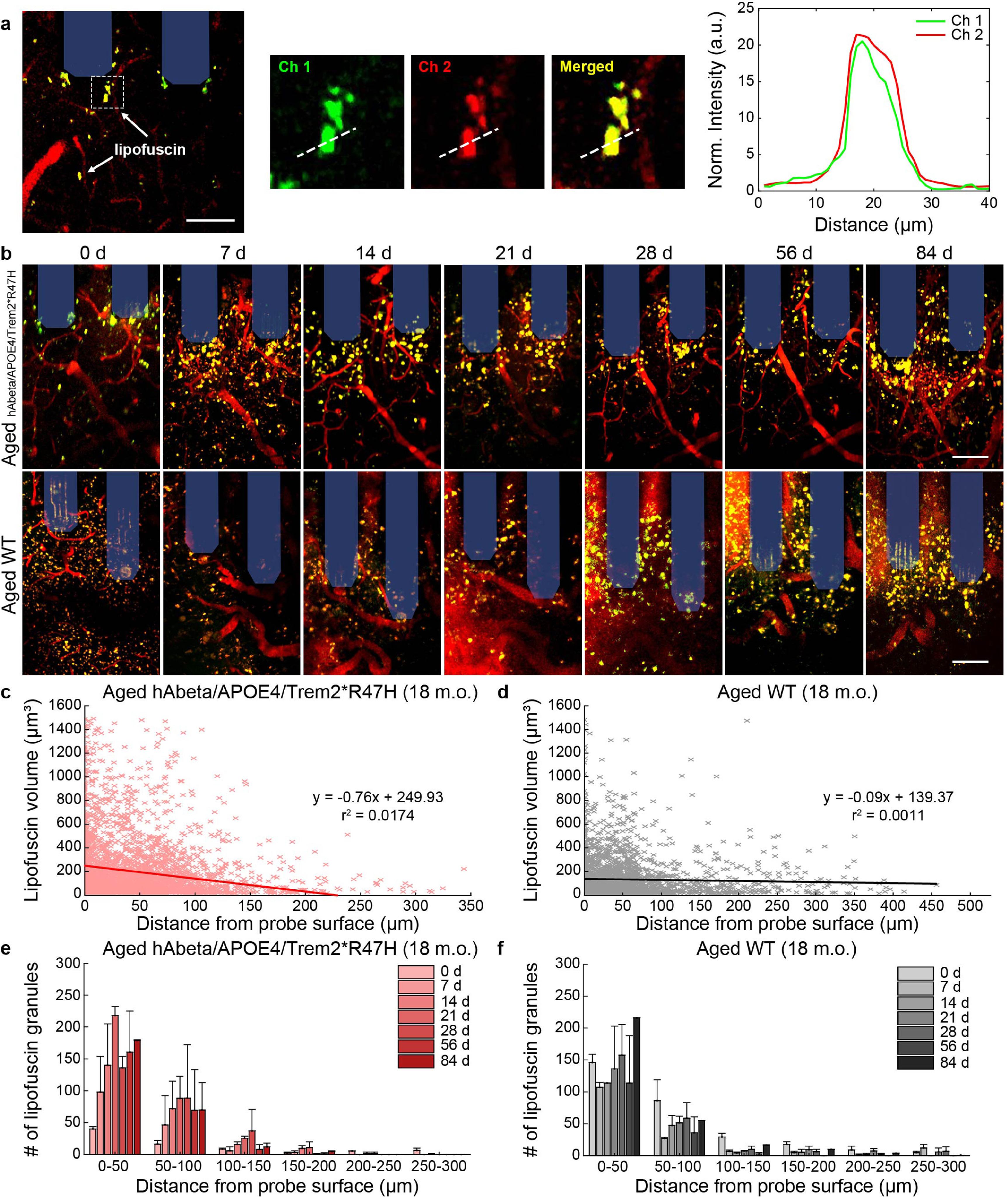
Lipofuscin accumulation around chronically implanted microelectrodes in aged WT and hAbeta/APOE4/Trem2*R47H mice. (a) Example two-photon image of lipofuscin signal detected across multiple wavelength emission filters. Lipofuscin can be detected by a 1:1 fluorescence intensity overlap in both red and green filtered channels. (b) Representative two-photon images of accumulated lipofuscin granules (*yellow*) around multi-shank microelectrode array (*shaded blue*) over 12 week post-implantation in aged (18 m.o.) WT and hAbeta/APOE4/Trem2*R47H mice. Scale bars = 50 *μ*m. (c)–(d) Scatter plots demonstrating trend in volume of lipofuscin granules with respect to distance from probe surface (aged hAbeta/APOE4/Trem2*R47H: 3173 lipofuscin granules across seven timepoints; aged WT: 2757 lipofuscin granules across seven timepoints). (e)–(f) Average number of lipofuscin granules with binned distance from the probe in aged hAbeta/APOE4/Trem2*R47H and WT mice (*n* = 2 mice per group). All data is reported as mean ± SEM.

Lipofuscin is an undegradable pigment with natural auto-fluorescent properties that can be imaged without the need for transgenic models or additional fluorophore labeling using 1:1 fluorescent signal detected across multiple wavelength emission filters (green and red channels) using two-photon microscopy (figure 2(a)), consistent with reports from other groups using similar methods to characterize lipofuscin appearance [33, 34]. Lipofuscin accumulates within the brain in an age-dependent manner, which we have confirmed by readily visualizing dual-fluorescent lipofuscin granules in the contralateral (unimplanted) cortex of 18 m.o. wild-type mice while younger 6 m.o. mice do not present any apparent lipofuscin pigments (supplementary figure 1). Lipofuscin forms when lysosomes are unable to fully degrade and recycle cellular waste due to insufficient pH and lysosomal hydrolysis [35–37]. To assess whether chronic microelectrode implantation leads to aberrant protein aggregation with aging or in neurodegenerative disease, lipofuscin was assessed over a 12 week implantation period in an 18 month-old AD mouse model (hAbeta/APOE4/Trem2*R47H). The hAbeta/APOE4/Trem2*R47H mouse is a triple mutant strain carrying a humanized *ApoE* knock-in mutation, a humanized *App* allele, and a *Trem2* mutation. Accumulation of lipofuscin was readily observed over time around an implanted microelectrode array in both the hAbeta/APOE4/Trem2*R47H model and an age-matched wild-type mouse (figure 2(b)). Lipofuscin granules increased in volume and accumulated in larger densities with closer proximity to the implant surface (figures 2(c)–(f)). These results demonstrate that chronic microelectrode arrays can exacerbate aberrant protein aggregation near the site of implantation.

### 3.2. Chronic electrode implantation halts the growth of pre-existing MX04-labeled plaques while promoting local accumulation of new MX04-labeled clusters

**Figure 3.**
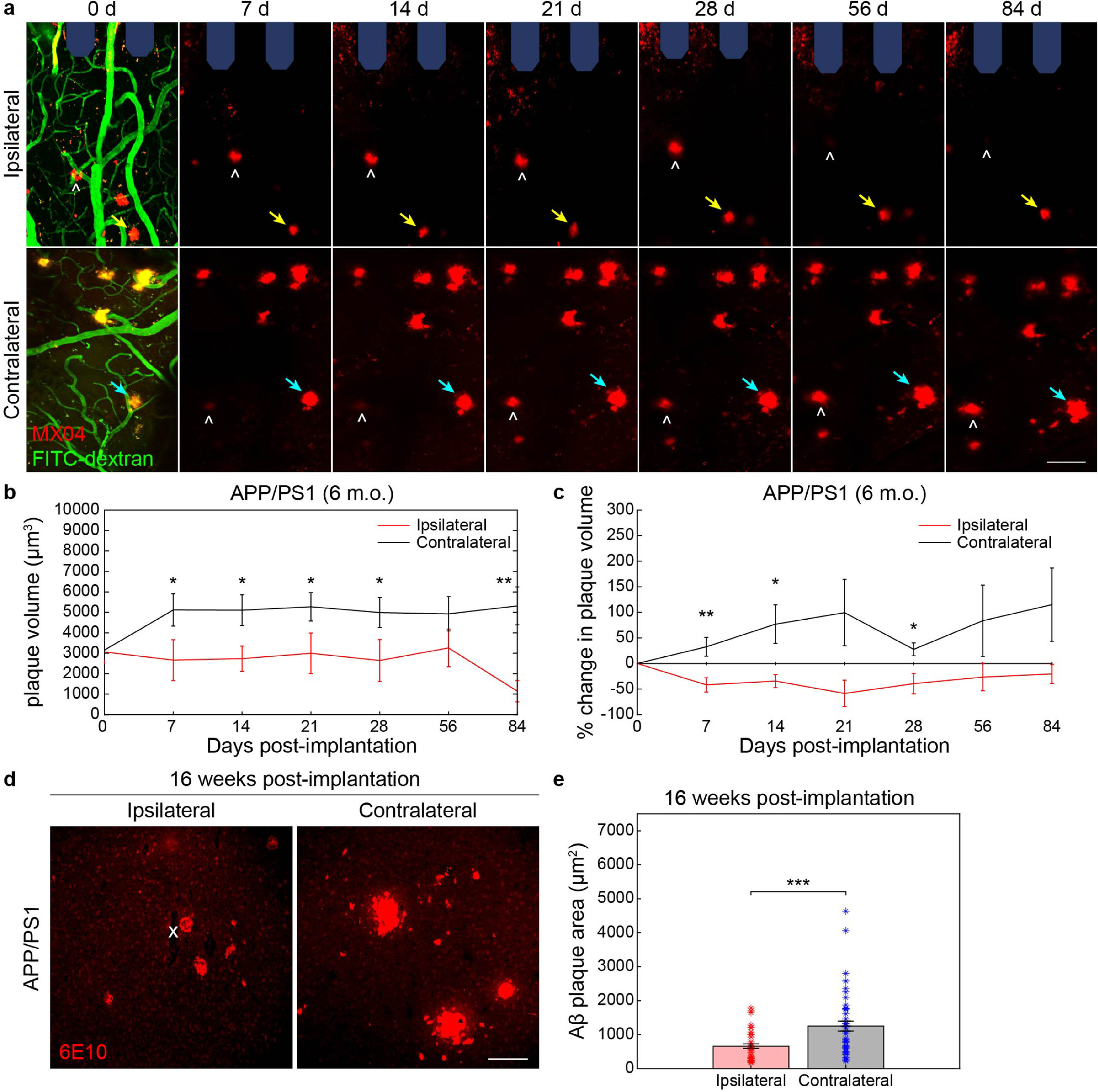
Chronic microelectrode implantation reduces the growth of local amyloid plaques in adult APP/PS1 mice. (a) Representative two-photon images of methoxy-X04 labeled plaques (MX04, *red*) and blood vessels (FITC-dextran, *green*) in ipsilateral hemisphere around multi-shank microelectrode array (*shaded blue*) over 12 weeks post-implantation in adult (6 m.o.) APP/PS1 mice compared to contralateral (uninjured) hemisphere. Ipsilateral hemisphere demonstrates plaques which do not visually change in size with chronic implantation (*yellow arrow*) whereas plaques on the contralateral hemisphere appear to increase in size over time (*cyan arrow*). NOTE: some plaques move into and out of frame over time (*white hat*) due to tissue drift between subsequent chronic imaging sessions. Scale bar = 50 *μ*m. (b) Change in plaque volume over a 12 week implantation period between ipsilateral and contralateral hemispheres in adult APP/PS1 mice (25 A*β* plaques on ipsilateral hemisphere and 27 plaques on contralateral hemisphere tracked longitudinally over seven time points across *n*= 3 mice). (c) Percent change in plaque volume with respect to plaque size on day 0 of electrode insertion over a 12 week implantation period between ipsilateral and contralateral hemispheres in adult APP/PS1 mice. (d) Representative immunohistology stain for 6E10, an A*β* marker, following 16 weeks post-implantation in adult (6 m.o.) APP/PS1 mice demonstrating visually reduced A*β* plaque sizes in ipsilateral hemisphere around the site of probe insertion (denoted by white ‘x’) compared to contralateral side. Scale bar = 100 *μ*m. (e) Average A*β* plaque area measured by 6E10 stain between ipsilateral and contralateral hemispheres (*n*= 42 A*β* plaques on ipsilateral hemisphere over 13 histological tissue sections and *n* = 45 A*β* plaques on contralateral hemisphere over 17 histological sections across 6 mice total). * *p* < 0.05, ** *p* < 0.01, *** *p* < 0.001. All data is reported as mean ± SEM.

A*β* and tau are other proteins, separate from lysosomes, whose aggregation and insufficient clearance from the brain are implicated in aging and neurodegenerative disease. To understand whether device implantation injury impacts the morphology of pre-existing plaques within the brain, we inserted microelectrodes within AD mice that are genetically designed to develop plaques and then longitudinally assessed the growth of nearby plaques using intravenously administered MX04 over a 12 week implantation period. The hAbeta/APOE4/Trem2*R47H mice used to assess lipofuscin accumulation did not exhibit any visible MX04-labeled plaques by 18 months of age, so we instead implanted microelectrodes in the cortex of APP/PS1 mice, which is a transgenic AD mouse model that naturally develops large, visibly identifiable plaques by 6 months of age (supplementary figure 2). APP/PS1 mice provided us the opportunity to assess changes in volume of pre-existing plaques around chronically implanted microelectrodes. The position and distribution of plaques within the tissue and with respect to other nearby plaques remained consistent over a 12 week imaging period, enabling accurate measurement of session-to-session changes in individual plaque volumes (figure 3(a)). In the uninjured cortex of APP/PS1 mice, plaques begin to manifest around 5 months of age and continually increase in size until around 10–12 months [22]. Fascinatingly, we reveal that the plaques located on the ipsilateral hemisphere around chronically implanted microelectrodes do not change in size with chronic implantation compared to plaques on the contralateral hemisphere, whose volumes were significantly increased (figure 3(b), *p <*0.05, two-way ANOVA). We also show that the plaques quantified on the ipsilateral hemisphere near implanted microelectrodes decrease in size over a 12 week implantation period whereas plaques distal from the electrode demonstrate a significant increase in percent change in volume during the implantation period (figure 3(c), *p <*0.05, two-way ANOVA).

In support of our *in vivo* imaging results, we demonstrate via immunohistochemical staining of post-mortem tissue for 6E10, a common AD marker used to label A*β*, that amyloid plaques on the ipsilateral hemisphere are considerably smaller than plaques found on the contralateral hemisphere following 16 weeks post-implantation in APP/PS1 mice (figure 3(d)). Quantifying the stained 6E10 area of individual A*β* plaques, we determine that plaques on the ipsilateral hemisphere are, on average, significantly smaller than those located further away on the contralateral hemisphere (figure 3(e)), *p*< 0.001, Welch’s *t-*test). Altogether, these findings suggest that device implantation injury effectively inhibits the growth of A*β* plaques located around chronically implanted microelectrodes.

**Figure 4.**
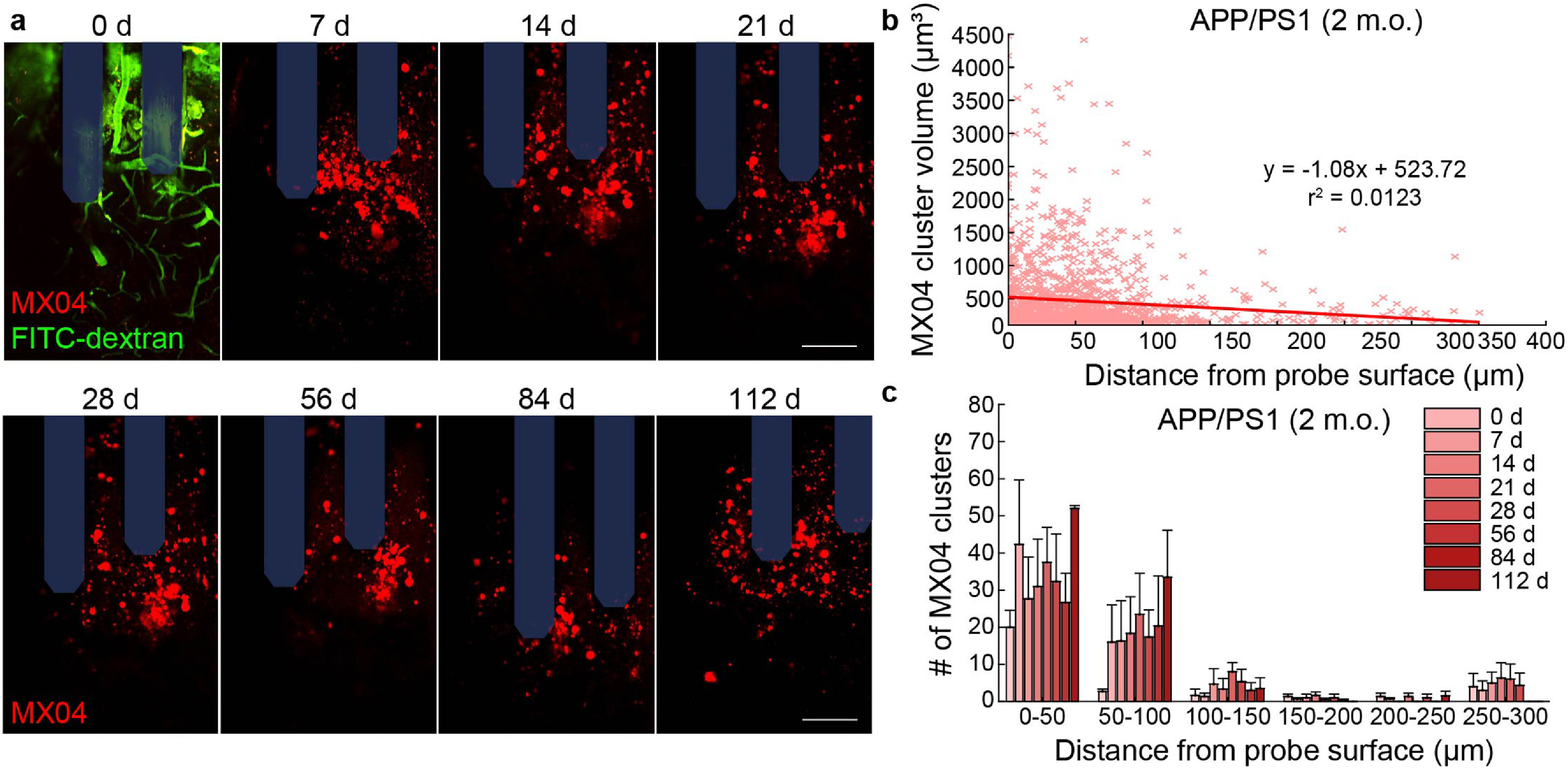
Accumulation of amyloid clusters around chronically implanted microelectrodes in young APP/PS1 mice. (a) Representative two-photon images of methoxy-X04 labeled clusters (MX04, *red*) and blood vessels (FITC-dextran, *green*) around multi-shank microelectrode array (*shaded blue*) over 16 weeks post-implantation in young (2 m.o.) APP/PS1 mice. Scale bars = 50 *μ*m. (b) Scatter plot demonstrating a trend in increased volume of MX04 clusters with respect to distance from probe surface (1407 MX04 clusters across seven timepoints). (c) Average number of MX04 clusters with binned distance from the probe in young APP/PS1 mice (*n* = 3). All data is reported as mean ± SEM.

To determine whether microelectrode implantation preferentially favors the accumulation of plaques, we first inserted microelectrode arrays within 2 month-old APP/PS1 mice. APP/PS1 mice at this age have yet to present stereotypical amyloid or tau pathology within the brain and therefore provide an opportunity to assess whether device injury triggers the aggregation of amyloid or tau protein prior to the temporally defined onset of neuropathology within the AD model. It is also important to note that these mice do not yet present lipofuscin pigments within the brain at this age as well. Surprisingly, we observed the appearance of MX04-labeled deposits, in which we term here as ‘MX04-labeled clusters’, within just the first 7 d following microelectrode implantation in young APP/PS1 mice (figure 4(a)). From our analyses, we reveal a slight negative association between the size of MX04-labeled clusters and their individual distances from the surface of an implanted microelectrode array (figure 4(b), *R* ^2^ = 0.0123). Additionally, we determined that the number of quantified MX04-labeled clusters increases preferentially with distance near the implant and over time following chronic microelectrode implantation (figure 4(c)). These results suggest that the tissue injury sustained from chronic electrode insertion within the brain accelerates the accumulation of Alzheimer’s pathology weeks to months before the expected onset of neuropathology within an uninjured mouse model of AD.

### 3.3. Elevated phagocytosis in activated microglia and APP in reactive astrocytes around chronically implanted electrodes

**Figure 5.**
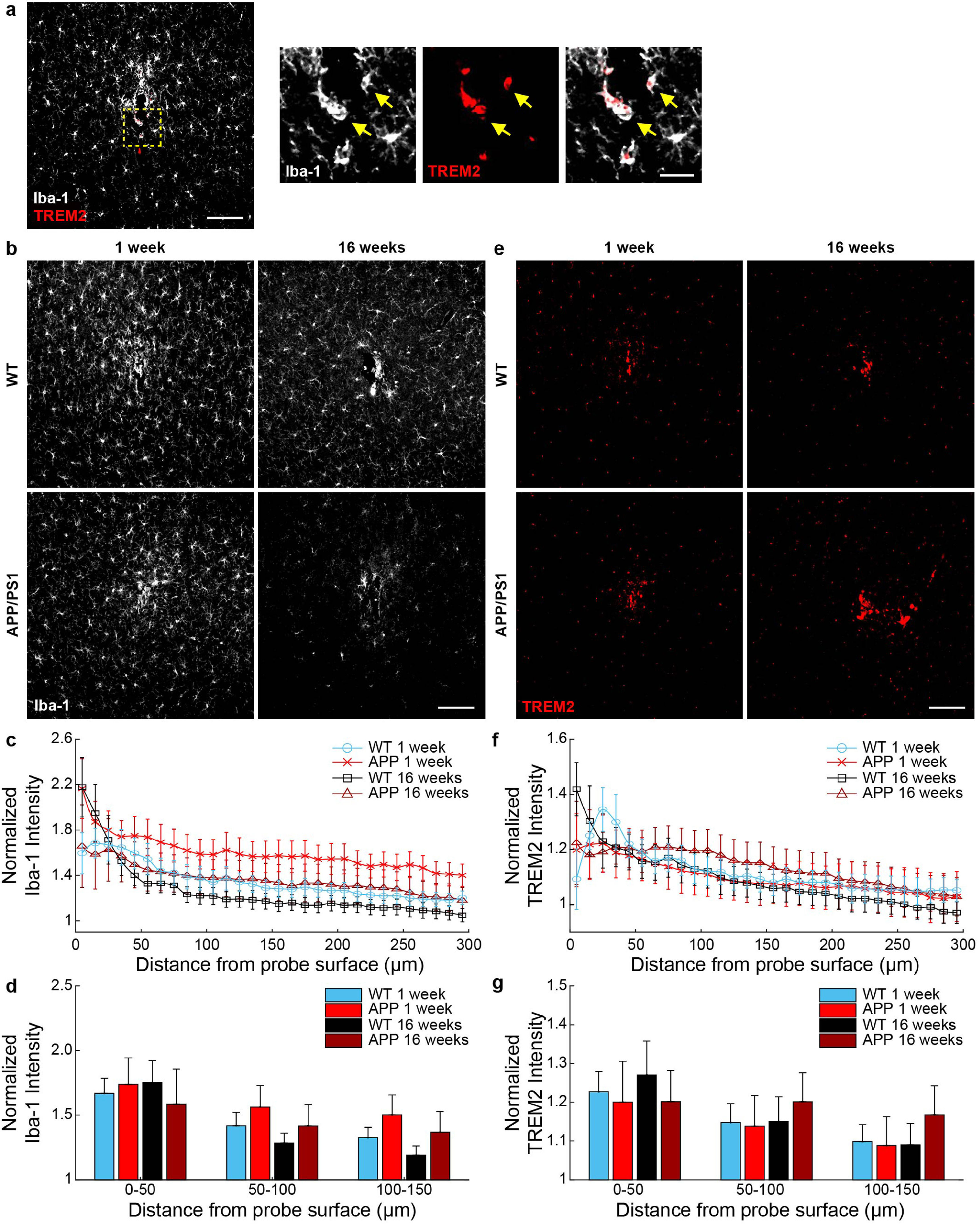
Microglial expression of phagocytic receptors around chronically implanted microelectrodes at 1 and 16 weeks post-implantation in WT and APP/PS1 mice. (a) Immunohistological representation demonstrating co-localization of Iba-1 + microglia (*white*) with triggering receptor expressed on myeloid cells 2 (TREM2, *red*) around the site of electrode implantation (*yellow arrows*). Scale bar = 100 *μ*m, 10 *μ*m (inset). (b) Representative images of Iba-1 fluorescence staining around implanted microelectrodes. Scale bar = 100 *μ*m. (c) Normalized Iba-1 fluorescence intensity with respect to distance from microelectrodes. (d) Average Iba-1 fluorescence intensity within 50 *μ*m bins up to 150 *μ*m around chronically implanted microelectrodes. (e) Representative images of TREM2 fluorescence staining around implanted microelectrodes. Scale bar = 100 *μ*m. (f) Normalized TREM2 fluorescence intensity with respect to distance from microelectrodes. (g) Average TREM2 fluorescence intensity within 50 *μ*m bins up to 150 *μ*m around microelectrodes (*n* = 6 mice per group at 1 week, *n* = 7 mice per group at 16 weeks). All data is reported as mean ± SEM.

Microglia assume a critical role in phagocytosing cellular and tissue debris following brain injury or amyloid protein in AD and their dysfunction has been linked to the progression of neurodegenerative disease [38, 39]. To determine whether phagocytosis within microglia due to device injury is elevated around implanted microelectrodes or within AD, explanted WT and APP/PS1 brain tissue were stained for Iba-1, a microglial marker, and ‘triggering receptor expressed on myeloid cells 2ʹ (TREM2), an innate immune receptor predominantly expressed by microglia within the brain during conditions of metabolic stress [25]. Notably, we found that microglia near the site of electrode implantation strongly co-localize with the expression of TREM2 (figure 5(a)). As expected, Iba-1 fluorescence intensities were visually elevated near the site of probe implantation at 1 and 16 weeks post-implantation in WT and APP/PS1 mice (figure 5(b)). While normalized Iba-1 fluorescence intensities were increased with proximity to the site of electrode implantation, we did not reveal any significant differences in Iba-1 fluorescence intensities between WT and APP/PS1 at either 1 week or 16 week post-implantation (figures 5(c) and (d)). Interestingly, we also observed a visual increase in TREM2 fluorescence intensities near the site of probe implantation at 1 and 16 weeks post-implantation in WT and APP/PS1 mice (figure 5(e)). While normalized TREM2 fluorescence intensities were increased closer to the site of implantation, we did not reveal any significant differences in TREM2 fluorescence intensities between WT and APP/PS1 at either 1 week or 16-week post-implantation (figures 5(f) and (g)). Nevertheless, these findings demonstrate that activated microglia increase expression of cellular immune receptors reportedly involved in phagocytosis of cellular debris and A*β* around chronically implanted microelectrodes.

**Figure 6.**
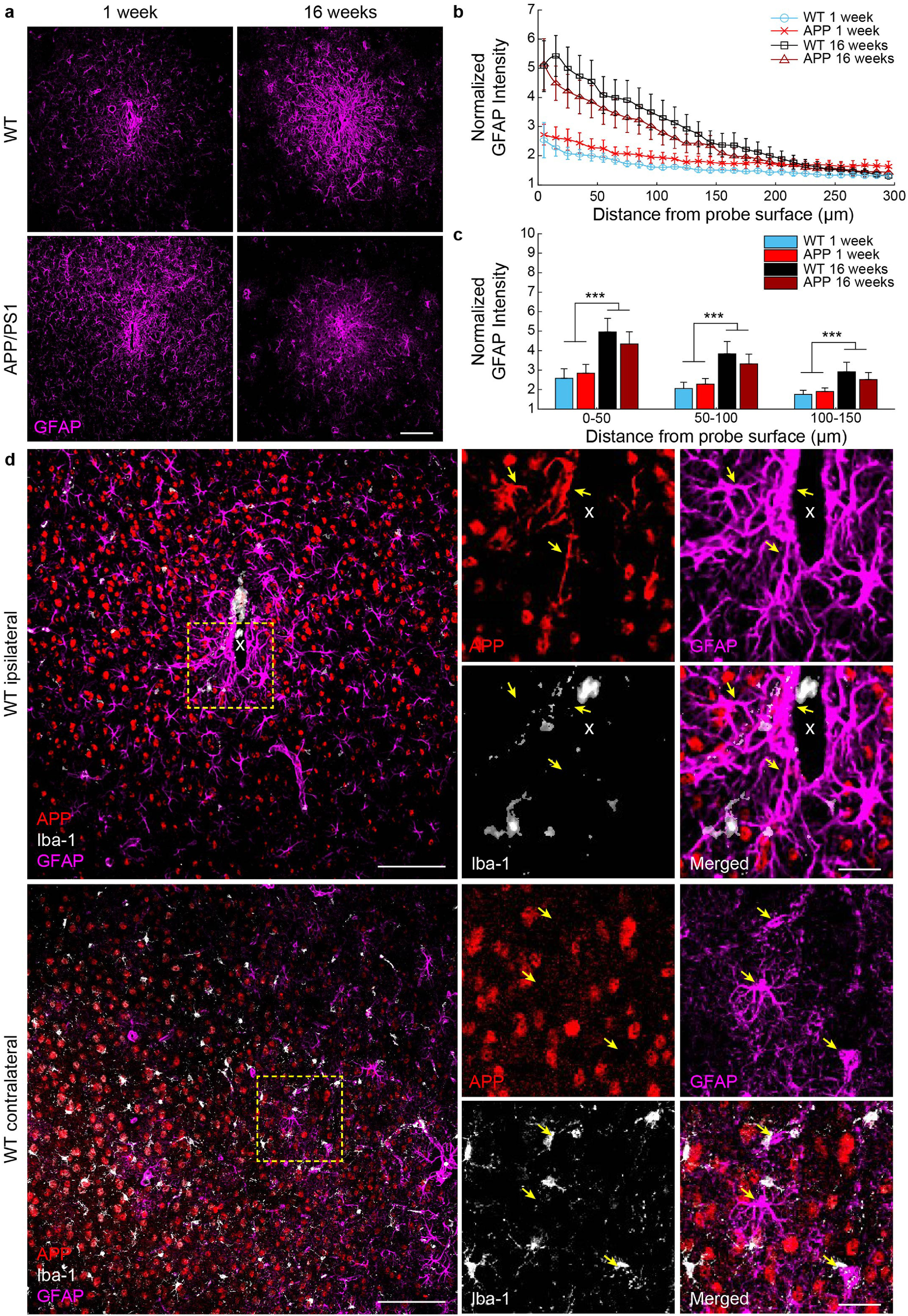
Reactive astrocytes express amyloid precursor protein around chronically implanted microelectrode arrays at 1 and 16 weeks post-implantation in WT and APP/PS1 mice. (a) Representative images of GFAP+ reactive astrocyte staining (*magenta*) around implanted microelectrodes. Scale bar = 100 *μ*m. (b) Normalized GFAP fluorescence intensity with respect to distance from the implanted microelectrode. (c) Average GFAP fluorescence intensity within 50 *μ*m bins up to 150 *μ*m around chronically implanted microelectrodes (*n*= 6 mice per group at 1 week, *n*= 7 mice per group at 16 weeks). (d) Immunohistological example demonstrating expression of amyloid precursor protein (APP, *red*) in GFAP+ astrocytes (*magenta*) but not Iba-1 + microglia (*white*) on the ipsilateral, but not contralateral hemisphere, near the site of a chronically implanted microelectrode in a WT mouse. Scale bar = 100 *μ*m, 25 *μ*m (inset). *** *p* < 0.001. All data is reported as mean ± SEM.

Separate from microglia, reactive astrocytes are the main culprits in formation of an astrocytic scar around intracortical electrodes and their dysfunction is commonly reported in neurological disorders [18, 26]. To assess whether astrocyte reactivity due to chronic microelectrode implantation is exacerbated in a mouse model of AD, explanted WT and APP/PS1 brain tissue were stained for GFAP, a marker for reactive astrocytes. As expected, GFAP+ astrocyte staining was markedly increased near the site of device implantation in both WT and APP/PS1 mice at 1 and 16 weeks post-insertion (figure 6(a)). GFAP fluorescence intensities were significantly elevated at 16 weeks post-implantation in both WT and APP/PS1 compared to 1-week post-implantation up to 150 *μ*m from the site of electrode implantation (figures 6(b) and (c)), *p*< 0.001, Welch’s *t*-test). Astrocytes reportedly can contribute to A*β* deposition due to their own production of APP [27, 28]. APP is a transmembrane protein whose pathological cleavage into aggregate forms of A*β* lead to the formation of senile plaques within AD and other dementia [29]. Intriguingly, we report that GFAP+ astrocytes express APP near the site of probe implantation (figure 6(d)). This co-expression of APP was specific to astrocytes and not microglia, since APP only co-labeled with GFAP+ astrocyte cells but not with Iba-1+ microglia cells. Additionally, co-labeling of APP+ and GFAP+ signal was only observed on the ipsilateral hemisphere, near the site of implantation, but not in contralateral (unimplanted) control tissue. These results suggest that device implantation injury can induce the expression of potential precursors for A*β* production within reactive astrocytes around chronically implanted microelectrodes.

### 3.4. Neuronal densities are reduced near chronically implanted microelectrodes

**Figure 7.**
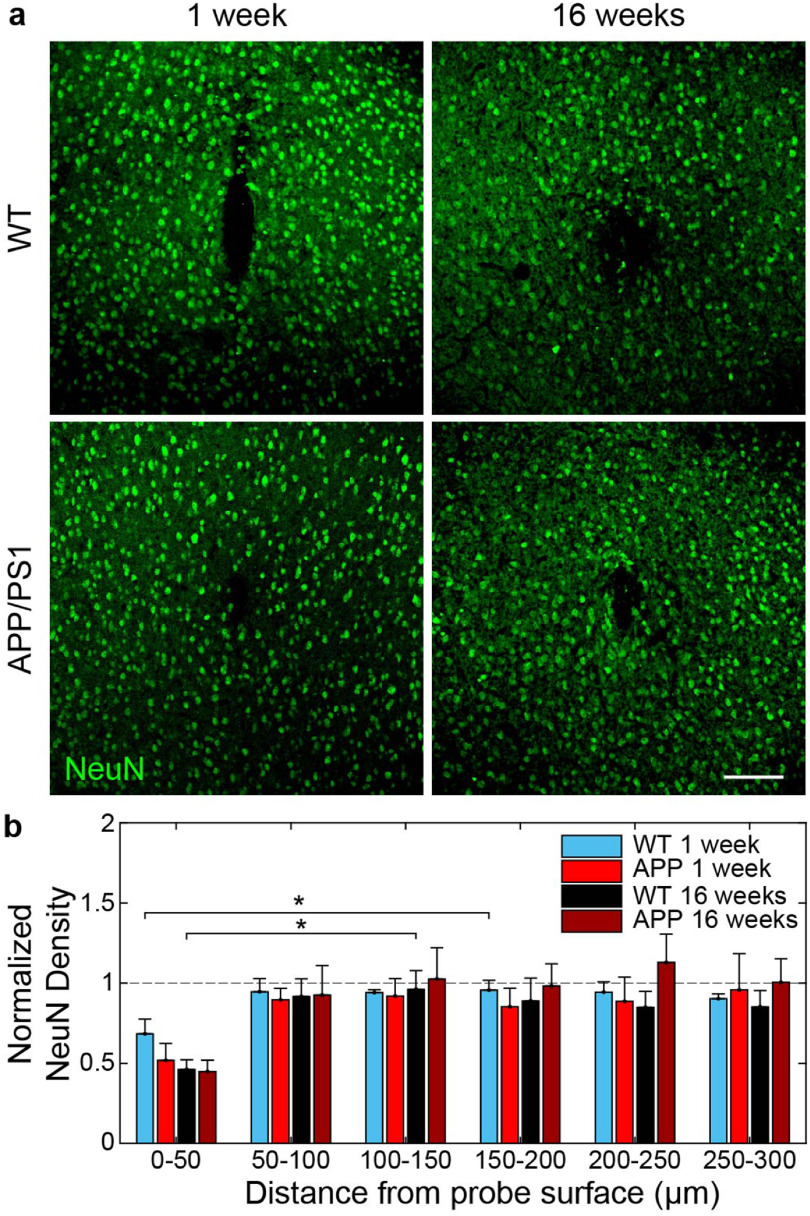
Reduced neuronal densities near chronically implanted microelectrodes at 1 and 16 weeks post-implantation in WT and APP/PS1 mice. (a) Histological representation of neurons (NeuN, *green*) around chronically implanted microelectrodes. Scale bar = 100 *μ*m. (b) Average NeuN density within 50 *μ*m bins up to 300 *μ*m around chronically implanted microelectrodes (*n*= 6 mice per group at 1 week, *n*= 7 mice per group at 16 weeks). * *p* < 0.05. All data is reported as mean ± SEM.

We previously reported that neuronal densities are affected around chronically implanted microelectrodes up to 4 weeks post-implantation [7]. Here, we stain for NeuN to visualize the distribution of neurons around microelectrode arrays in WT and APP/PS1 mice at 1 and 16 weeks post-implantation (figure 7(a)). Quantification of neuronal densities normalized to contralateral hemispheres demonstrated a decrease in neuronal densities within the 0–50 *μ*m region near the site of implantation in both WT and APP/PS1 mice (figure 7(b)). Specifically, we determined that neuronal densities were significantly reduced in tissue regions between 0–50 *μ*m and 100–150 *μ*m at 1 week post-implantation and between 0–50 *μ*m and 50–100 *μ*m at 16 weeks post-implantation in WT mice but not APP/PS1 mice (*p*< 0.05, two-way ANOVA). However, we did not detect any significant differences in neuronal density between WT and APP/PS1 mice at either 1 or 16 week post-implantation (*p >*0.05, two-way ANOVA). Therefore, these findings suggest that acute and chronically implanted microelectrodes result in the loss of neurons local to the site of implantation regardless of the animal’s genetic susceptibility to disease.

### 3.5. Abnormally phosphorylated tau marks regions of axonal and myelin loss around chronically implanted electrodes

**Figure 8.**
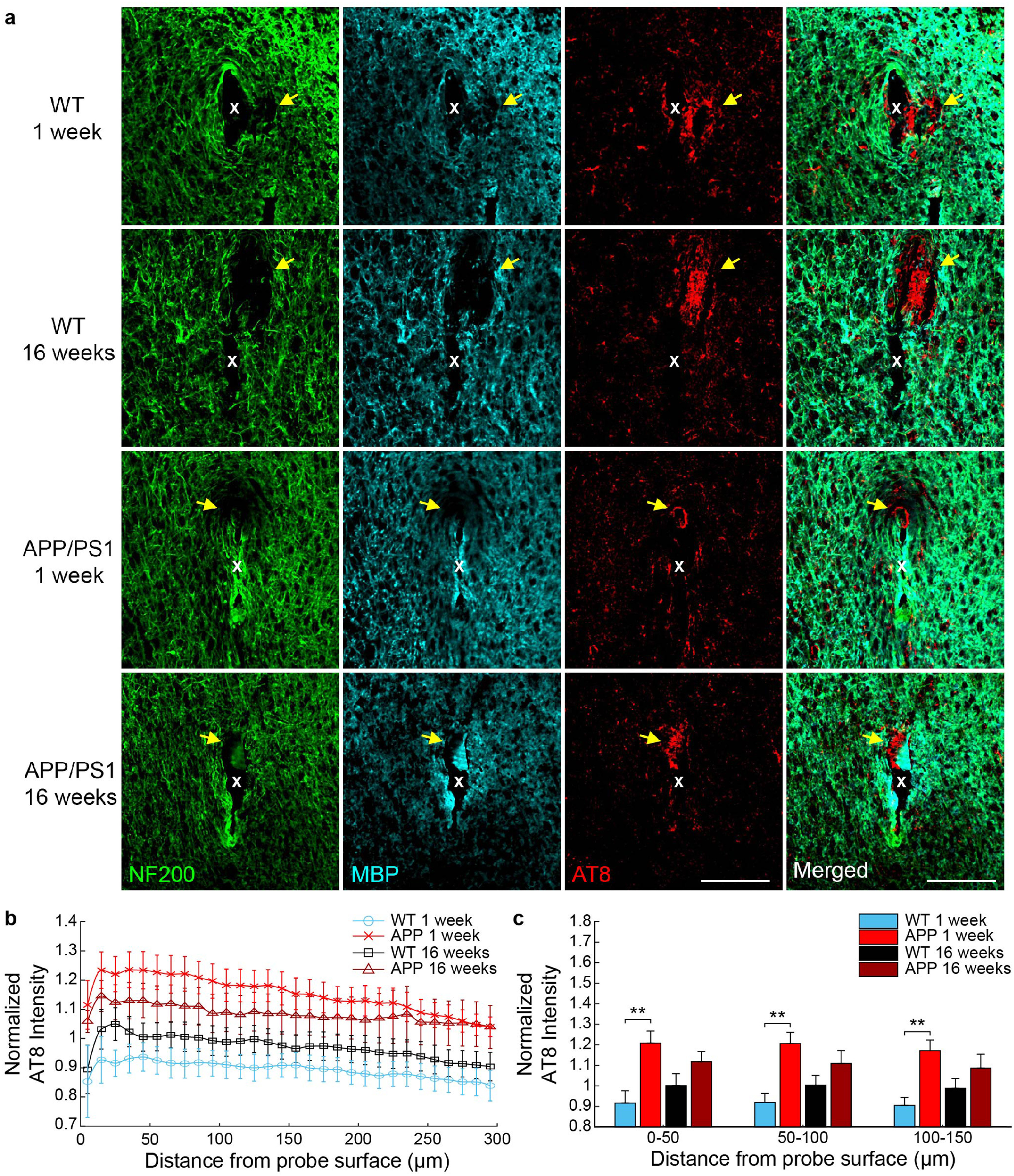
Axon and myelin pathology is associated with abnormal tau phosphorylation around chronically implanted microelectrodes at 1 and 16 weeks post-implantation in WT and APP/PS1 mice. (a) Immunostaining for axons (NF200, *green*), myelin (MBP, *cyan*), and phospho-tau (AT8, *red*) reveals abnormal tau phosphorylation in areas of axon and myelin loss (*yellow arrows*) around 1 and 16 week implanted probes (white ‘x’) in WT and APP/PS1 mice. Scaler bar = 100 *μ*m. (b) Normalized AT8 fluorescence intensity with respect to distance around chronically implanted microelectrodes. (c) Average AT8 fluorescence intensity within 50 *μ*m bins up to 150 *μ*m around chronically implanted microelectrodes (*n* = 6 mice per group at 1 week, *n* = 7 mice per group at 16 weeks). ** *p* < 0.01. All data is reported as mean ± SEM.

Previously, we demonstrated that chronic electrode implantation leads to the re-organization of axonal and myelin fibers near the site of probe implantation [18, 30]. In this study, we aimed to better understand the pathology governing axonal and myelin loss during device implantation injury and whether there is an impact following microelectrode implantation in a mouse model of AD. Staining explanted brain tissue with NF200, an axonal protein, and MBP, for myelin basic protein, we reveal unevenly distributed areas of axonal and myelin loss with close proximity to the site of microelectrode implantation (figure 8(a)). Interestingly, when we co-stain for AT8, a marker commonly used to study hyperphosphorylated tau in neurodegenerative disease [31], we reveal that these areas of axon and myelin loss (i.e. NF200- and MBP- signal) correspond with abnormally high levels of AT8+ phosphorylated tau. AT8 staining intensity is visually increased near the site of probe implantation (figure 8(a)). When observing similar staining patterns in contralateral control tissue, we do not observe abnormal patterns of aggregated phosphorylated tau nor do we observe loss of axonal or myelin integrity, suggesting this is an injury-specific phenomenon (supplementary figure 3). Normalized fluorescence intensities of AT8 are increased above baseline levels near the site of electrode implantation in APP/PS1 mice, but not WT mice, at 1 and 16 weeks post-implantation (figure 8(b)). The average normalized AT8 fluorescence intensity was significantly increased in APP/PS1 mice at 1-week post-implantation compared to WT mice, but not at 16 weeks post-implantation (figure 8(c), *p* < 0.01, Welch’s *t*-test). These results suggest that axonal and myelin pathology following device implantation injury is associated with abnormal phosphorylation of tau.

## 4. Discussion

The aim of this study was to understand whether the brain’s immune response to neural electrode technology mimics neuropathology typically observed with neurodegenerative disease. Neuroinflammation, glial activation, and vascular dysfunction are all characteristic tissue events shared between both chronic implants and degenerative brain diseases, such as AD. The neuropathology of AD has been studied for more than a century, much longer than that of the foreign body response to neural microelectrodes. As a result, common biomarkers classically observed in disease, such as A*β* and neurofibrillary tau tangles, have been identified and implicated in the development of neurodegeneration and chronic inflammation within diseased brains. Therefore, it would be of interest to determine whether any of the identified hallmarks of neurodegeneration in AD are also involved in the biological failure mode of chronically implanted microelectrodes within the brain. Identifying predominant cellular and tissue mechanisms compromising neural health and neurotransmission around recording and stimulating brain implants will aide in the discovery and innovation of therapeutic approaches to improve the fidelity and longevity of neural electrode interfaces.

### 4.1. Accumulation of age-related lipofuscin granules following chronic electrode implantation

With aging and cellular senescence comes a natural decline in cellular metabolism, leading to the buildup of harmful metabolic waste. Lipofuscin, an auto-fluorescent composite of proteins, lipids, and sugars, accumulates naturally within the brain with age [40]. These lipofuscin granules are primarily found within lysosomes of post-mitotic and senescent cells, suggesting they are unable to be degraded normally through cellular metabolism [41, 42]. It is believed that this loss of lysosomal hydrolysis is due to decreases in lysosomal acidity and metabolic supply to sustain lysosome pH [35–37]. Furthermore, it has been reported that brain injury can exacerbate neuroinflammation and neurodegeneration associated with lipofuscin accumulation [24]. We report here that chronically implanted microelectrodes promote lipofuscin aggregation within the aged brain (figure 2).

Lipofuscin accumulation around microelectrodes occurred in both WT and AD mice alike, although there was an increased trend for AD mice to produce lipofuscin deposits that were greater in both number and size within the electrode microenvironment. An impairment in the clearance and removal of pathological proteins and cellular waste is a commonly proposed theory of neurodegeneration within both natural aging and brain disease. The functional role of lipofuscin deposits within the brain and whether they are neurotoxic remains poorly understood. However, lipofuscin accumulation has been previously associated with cellular oxidative stress as well as impairments in memory function, suggesting they have some impact on the health of neuronal tissue and regulation of brain activity [43]. Elevated accumulation of lipofuscin granules observed here around chronically implanted microelectrodes suggests that electrode implantation injury induces a backstop in the clearance mechanisms regulating removal of cellular debris and metabolic waste from the brain. However, we could not determine the cellular origin of lipofuscin from our two-photon data. Furthermore, lipofuscin has been reported in glial cells as well as neurons [24, 44–46], although it is not clear whether this is due to accumulation of lipofuscin in aged or dying cells or due to glial-uptake of cellular debris. We have previously reported that microelectrode implantation leads to disruption of not only neurons but non-neural cells as well [47]. Future work should focus on identifying the cellular culprits responsible for depositing this metabolic by-product as well as the functional consequences on surrounding neural tissue and long-term functional device performance.

### 4.2. Patterns of plaque deposition following chronic electrode implantation

Our initial hypothesis was that chronic microelectrode implantation would exacerbate the growth of pre-existing plaques either due to elevated deposition of neuronal or glial A*β*/tau or due to an impairment in glial clearance of amyloid or tau. Contrary to this hypothesis, we observed that plaques existing prior to the start of electrode implantation were, on average, smaller in volume over a 12 week implantation period compared to plaques located further away in distal brain regions (figure 3). This was the consequence of an overall negative change in size of plaques around microelectrodes. In contrast, plaques on the contralateral, uninjured hemisphere gradually increased in size due to the natural progression of neuropathology within this mouse model.

One possible explanation is that an increase in plaque size on the ipsilateral hemisphere was impaired due to a degradation in chronic imaging quality through an optical window around implanted microelectrodes over time, which would present a confound in our findings. However, in support of our two-photon findings, we observed a similar decrease in the overall area of plaques via post-mortem immunohistochemical staining confirming that the microenvironment around chronically implanted microelectrodes halts the rate of plaque growth (figures 3(d) and (e)). It is well understood that glial cells are responsible for the degradation and clearance of amyloid and tau from the brain and that their physiological dysfunction in AD could lead to the aberrant accumulation of aberrant proteins, eventually forming senile plaques [48, 49]. One possible interpretation of findings could be that an enhanced glial response results in the reduction in growth of nearby plaques. We show here that both microglia and astrocyte activity is upregulated with respect to chronically implanted microelectrodes in both WT and APP/PS1 mice alike (figures 5 and 6).

Despite our results demonstrating that microelectrodes stall the growth of plaques locally, we also report novel plaque deposition proximal to the electrode-tissue interface (figure 4). The MX04-labeled clusters we observed were smaller in size compared to traditionally measured plaques yet accumulated in greater numbers throughout the course of implantation. The morphology of these clusters also appeared rounder, denser, and more symmetrical in shape, different than that of plaques which are commonly asymmetrical and consist of a dense plaque core surrounded by diffuse amyloid fibrils [39]. Microglia are known to form a neuroprotective barrier around amyloid plaques, and denser, more compact plaques are generally found to have more microglia contact [50]. One interpretation is that these MX04-labeled clusters appear more compacted near the electrode where there is also an elevated microglial immune response and therefore more microglia association with amyloid. Alternatively, brain injury is known to increase the rate of APP processing, production of amyloid/tau, and aggregation of plaques [51, 52] and microglia naturally phagocytose and sequester amyloid for proper degradation and removal from the brain [48]. Therefore, another possible explanation could be that these MX04-labeled clusters do not represent typical extracellular plaque deposits but represent accumulation intracellularly within neurons and glial cells following implantation injury from chronic microelectrodes. Future studies discerning the origin of these pathological amyloid/tau by-products could reveal additional insight on the ongoing neurodegenerative processes around neural microelectrodes.

### 4.3. Glial basis for phagocytosis and generation of APP following electrode implantation

Microglia are the main potentiators of neuroinflammation both around chronic brain implants and with neurodegenerative disease. We report here that microglia activation is increased around chronically implanted microelectrodes in both WT and AD mice (figure 5). Also, we demonstrate that microglia close to the site of electrode implantation exhibit increased expression of Triggering receptor expressed on myeloid cells 2 (TREM2). TREM2 is an important metabolic receptor expressed predominantly within microglia and assists with critical microglial functions such as phagocytosis and removal of pathological waste. This receptor has been identified as a significant risk factor in the development of AD. It is suggested that dysfunction in this critical immune cell receptor is what impairs the ability for microglia to remove amyloid and tau from Alzheimer’s brains [39].

We demonstrate here that lipofuscin waste accumulates around chronically implanted microelectrodes in both WT and AD mice, but that AD mice trend more negatively (i.e. lipofuscin appears larger nearer to the electrode). Indeed, the hAbeta/APOE4/Trem2*R47H mouse model used in this study presents a mutation in the *Trem2* gene, potentially suggesting that increased lipofuscin accumulation around chronic microelectrodes within these mice is the result of impaired TREM2 function. Furthermore, impairment in TREM2 function interferes with the ability for microglia to form a protective barrier around amyloid plaques leading to increases in amyloid deposition and an increase in neuronal and axonal dystrophy [39]. It is also worth noting that microglia also have impaired ability to form a protective barrier around APOE4 used in this study compared to APOE3 [53]. APOE4 is a major genetic risk factor for AD and is implicated in multiple neurodegenerative processes within the brain, such as amyloid/tau accumulation, neuroinflammation, impaired synaptic plasticity, vascular dysfunction, and metabolism [54]. It is unclear from the results reported here whether TREM2 or APOE function is compromised around chronically implanted microelectrodes or within AD brains. Future studies would benefit from examining the functional purpose of TREM2 and APOE within microglia at the electrode-tissue interface and how their upregulation of this phagocytic receptor contributes to the foreign body response to chronically implanted microelectrodes.

Astrocytes mediate the formation of a neurochemically impermeable and neurotoxic glial scar as part of the foreign body response to chronic brain implants. Previously, we presented a dynamic spatiotemporal pattern of astrocyte reactivity within the first few weeks during implantation using two-photon imaging around implanted microelectrodes [10]. Here, we demonstrate that astrocyte reactivity significantly increases from 1 to 16 weeks post-implantation in both WT and AD mice (figure 6). Additionally, we report astrocyte-specific upregulation of APP in proximity to chronically implanted microelectrodes within WT mice. Based on these results, it is possible that the Increase in amyloid deposition observed around microelectrode implants could be partially due to A*β* generation from reactive astrocytes. APP is a transmembrane protein expressed on cellular members and cleaved by secretases. With proper cleavage, the amyloid derived from APP does not aggregate into A*β* plaques and can be easily removed from the brain. Improper cleavage of APP, such as with—secretases, produces an aggregated form of A*β* resulting in the pathological formation of senile plaques. Future research should be performed assessing the function role of APP expression within reactive astrocytes around chronically implanted microelectrodes.

### 4.4. Neuronal and axonal pathology following chronic electrode implantation

The presence of nearby healthy neurons is critical to the long-term performance of neural microelectrode arrays. Neural densities are characteristically reduced both acute and chronically with close spatial proximity to implanted microelectrodes [55–57]. In this study, we confirm that microelectrodes impact the number of neurons located adjacent to the site of implantation (figure 7). Despite a visible difference in the density of neurons within the 0–50 *μ*m tissue region around chronically implanted microelectrodes at 1 and 16 weeks post-implantation, we did not detect any significant differences between WT and APP/PS1 mice. It is important to keep in mind that at 16-weeks post-implantation APP/PS1 mice are at 6 months of age and neuronal numbers are not expected to be naturally altered within the AD model at this age [58–60]. Furthermore, our analyses are only limited to understanding whether NeuN+ neurons are present or not around the electrode compared to intact control regions but does not discern whether the neurons that remain are viable or whether they may be otherwise functionally impaired around chronically implanted devices. In support of this, there have been reports of explanted tissue populated by many neurons yet still demonstrating poor electrophysiological recording performance [5, 61]. One possible explanation is that the neurons which were not directly impacted by implantation injury are quiescent or their activity is somehow suppressed [5]. It is also important to note that trauma to the brain can alter the expression of neuronal proteins traditionally used to evaluate neuronal health and viability, such as NeuN, in the absence of any apparent neurodegeneration [62]. Therefore, further *in vivo* evaluation is needed to fully understand the physiological consequences imparted on neurons around chronically implanted microelectrodes and in rodent models of neurodegenerative disease.

Maintaining axonal and myelin integrity around chronically implanted electrodes is essential for effective transmission of neuronal information and excitability of neurons in brain regions both near and far from intracortical recording and stimulating electrodes. We have previously demonstrated re-organization and potential sprouting of axons as well as spatiotemporal patterns of axonal and myelin blebbing around intracortical electrodes [5, 7, 8]. Here, we demonstrate that electrode implantation can induce regions of axonal and myelin loss with proximity to the site of electrode insertion (figure 8). A previous study investigating local markers of tissue inflammation around chronically implanted microelectrodes noted the appearance of hyperphosphorylated tau at the lesion border within explanted brain tissue following 16 weeks of electrode implantation in rats [25]. This same study also noted similar neuropathology around the site of device insertion following 5 months of implantation in a human Parkinsonian patient, suggesting this phenomenon can occur in both rodents and humans. In line with this study, we observe elevated expression of phosphorylated tau near chronically implanted microelectrodes at 1 and 16 weeks post-implantation in both WT and AD mice. To our surprise, these areas of increased tau phosphorylation overlap with identified tissue regions lacking axons and myelin, suggesting that abnormal tau phosphorylation is an indicator of axonal and myelin pathology around chronically implanted microelectrodes. Phosphorylation of tau is a naturally occurring phenomenon important for microtubule assembly, axonal transport, and neuronal plasticity [63–65]. Hyperphosphorylated tau, however, is a pathological hallmark of AD in which misfolded tau proteins aggregate and form neurofibrillary tau tangles within axons, interfering with neuronal information transmission [65]. It is unclear from these findings whether hyperphosphorylated tau is the symptom or cause of axonal and myelin pathology around chronically implanted microelectrodes or whether abnormal tau phosphorylation would have a significant impact on the recording or excitability of neurons at the electrode-tissue interface. We have demonstrated previously that device implantation can lead to local demyelination and that demyelination on its own can directly affect functional recording performance of intracortical electrodes [8, 66, 67]. Furthermore, there is an emerging association between myelin dysfunction and AD-like pathology [23]. Understanding if tau phosphorylation precedes axonal and myelin loss (or vice versa) and if abnormal tau phosphorylation directly contributes to functional electrode performances could point towards novel biological targets to address neurodegeneration around chronic brain implants.

### 4.5. Future directions

While the pathological consequence of lipofuscin remains a mystery, it demonstrates no useful physiological purpose to date. Previous work has demonstrated that chronic treatment with the antioxidant melatonin can reduce lipofuscin content within the rat hippocampus [68]. Melatonin has previously shown to improve both microglia activation and recording performance of chronically implanted electrodes [69, 70]. Therefore, one way to address aberrant lipofuscin accumulation could be through the facilitation of glial function, such as enhancing efficient removal of cellular debris, reducing neuroinflammation, and promoting neuroprotection. APP itself is important for neural stem cell proliferation and axon outgrowth following injury and therefore may be neuroprotective in certain pathological conditions [71, 72]. However, impaired cleavage of APP by *β*-secretases generates precursors for A*β* deposition. It is currently unclear how the levels of *β*-secretase, as well as *α*- and *γ*-secretase, are impacted around chronically implanted microelectrodes. Finally, previous and current FDA-approved clinical trials have focused on improving cognitive and behavioral outcomes in AD by using monoclonal antibodies to target and promote clearance of A*β* plaques from the brain, yet the results have either been mixed or unsuccessful thus far [17, 73].

There is current debate on whether the presence of amyloid is a cause or solely a symptom of neurodegeneration within AD (i.e. ‘amyloidogenic’ vs. ‘non-amyloidogenic’ hypothesis) [74]. In either case, the abnormal accumulation of lipofuscin, APP, amyloid, and tau around chronically implanted electrodes indicates a significant failure in tissue clearance mechanisms and metabolic stress. These observations suggest that other harmful factors, such as reactive oxidative species and misfolded tau proteins, may also accumulate and pose a risk to neuronal health and function. It is important for future studies to investigate the potential accumulation of other tissue factors at the electrode-tissue interface to develop a more comprehensive understanding of the potentially detrimental biochemical processes occurring around chronically implanted electrodes.

Tau phosphorylation is mediated by a number of different kinases, such as cyclin-dependent kinase 5 (cdk5) and glycogen synthase kinase-3 (GSK-3) [75, 76], and phosphatases, such as protein phosphatase PP1, PP2A, and PP5 [77]. Accounting for the changes in expression levels of these critical regulators of tau phosphorylation around microelectrodes could point toward novel targets during neurodegeneration surrounding chronic electrode implantation. There is also evidence that the presence of amyloid can alter tau phosphorylation [78, 79]. It is important to note that the methoxy-X04 label used in this study binds to both amyloid and tau within the brain [80, 81]. Therefore, it is unclear whether the methoxy-X04 labeling around implanted microelectrodes is completely representative of amyloid protein or if there is also partial labeling of tau as well. Phosphorylated tau was also reported here as being increased in unusual densities around chronically implanted microelectrodes. However, it is worth pointing out that amyloid-β and tau are both present in Alzheimer’s plaques [32, 82, 83]. Future research should use more targeted investigative methods, such as using P130S mouse models more directed at studying tau pathology in AD, to disentangle the potential relationship between amyloid and tau at the electrode-tissue interface.

Overall, we did not report much significance in the difference between WT and AD mice for some of the patterns of neuropathology commonly shared between the two models. For two-photon imaging experiments, mice were aged to 6 months prior to electrode implantation to understand the impact of electrode implantation injury on the rate of growth of nearby pre-existing plaques. For histology experiments, APP/PS1 were implanted at 2 months of age and, by 16 weeks of implantation, mice were only 6 months old. At this age, APP/PS1 mice are just beginning to present amyloid deposition and therefore it is still relatively early in the progression of AD pathology typical for this mouse model. It could be, when comparing AD mice of a year or more in age to similarly age-matched controls, that we may observe a much greater difference in brain tissue responses following chronic microelectrode implantation. Despite this, we were still able to show that microelectrode implantation accelerates the onset of amyloid pathology even within 2 month-old APP/PS1 mice. Additionally, we reported novel findings on the expression of various cell and tissue factors related to aging and neurodegenerative disease around implanted microelectrodes which will aide future investigators in narrowing their research focuses and develop more informed intervention strategies for addressing the foreign body reaction to chronic brain implants.

## 5. Conclusion

The power of neural interface technology to aide in the discovery of previously unknown neuroscientific phenomenon and provide means for effective clinical therapy of neurological dysfunction is limited due to yet unresolved tissue responses to implanted recording and stimulating electrodes within the brain. These presently elusive biological mechanisms impairing the long-term application of neural microelectrodes could potentially be revealed by referencing the study of other brain disorders similarly plagued by neuronal, glial, and vascular dysfunction, such as AD. In this study, we employed mouse models of AD to reveal the change in expression of various tissue factors that offer new insights on neurodegeneration surrounding chronic brain implants. We determined that microelectrode implantation preferentially favors the accumulation of pathological proteins related to aging and neurological disease at the electrode-tissue interface. We also highlighted novel spatiotemporal patterns of glial-specific expression of different factors related to deposition and clearance of amyloid within the brain as well as new information on potential neurodegenerative mechanisms governing axonal pathology around chronic microelectrodes. In summary, these research findings provide a new perspective regarding the progression of neurodegenerative injury surrounding neural interface technology and will aide in more rigorous study of electrode-tissue biocompatibility as well as expedited development of advanced therapies for both brain injury and disease.

## Data Availability

The data that support the findings of this study are openly available at the following URL/DOI: bioniclab.org.
